# The Trends in Atrial Fibrillation-Related Mortality before, during, and after the COVID-19 Pandemic Peak in the United States

**DOI:** 10.3390/jcm13164813

**Published:** 2024-08-15

**Authors:** Inon Dimri, Ariel Roguin, Nashed Hamuda, Rami Abu Fanne, Maguli Barel, Eran Leshem, Ofer Kobo, Gilad Margolis

**Affiliations:** 1 Department of Cardiology, Hillel Yaffe Medical Centre, Hadera 3820302, Israel; yinond@hymc.gov.il (I.D.); ramia@hymc.gov.il (R.A.F.); eranl@hymc.gov.il (E.L.); 2 The Ruth and Bruce Rappaport Faculty of Medicine, Technion Israel Institute of Technology, Haifa 3525433, Israel

**Keywords:** COVID-19, cardiovascular mortality, atrial fibrillation

## Abstract

**Background**: During the first months of the COVID-19 outbreak, an increase was observed in atrial fibrillation (AF)-related mortality in the United States (U.S). We aimed to investigate AF-related mortality trends in the U.S. before, during, and after the COVID-19 pandemic peak, stratified by sociodemographic factors. **Methods**: using the Wide-Ranging Online Data for Epidemiologic Research database of the Centers for Disease Control and Prevention, we compared the AF-related age-adjusted mortality rate (AAMR) among different subgroups in the two years preceding, during, and following the pandemic peak (2018–2019, 2020–2021, 2022–2023). **Result**: By analyzing a total of 1,267,758 AF-related death cases, a significant increase of 24.8% was observed in AF-related mortality during the pandemic outbreak, followed by a modest significant decrease of 1.4% during the decline phase of the pandemic. The most prominent increase in AF-related mortality was observed among males, among individuals younger than 65 years, and among individuals of African American and Hispanic descent, while males, African American individuals, and multiracial individuals experienced a non-statistically significant decrease in AF-related mortality during the pandemic decline period. **Conclusions**: Our findings suggest that in future healthcare crises, targeted healthcare policies and interventions to identify AF, given its impact on patients’ outcomes, should be developed while addressing disparities among different patient populations.

## 1. Introduction

The outbreak of the coronavirus disease 2019 (COVID-19) pandemic created some indirect effects that significantly impaired the management of cardiovascular diseases [1]. The lockdown and the decrease in ambulatory procedures during the pandemic caused delays in the diagnosis and treatment of many cardiovascular diseases [2,3,4]. Concurrently, an increase was observed in cardiovascular mortality during the first months of the COVID-19 eruption in the U.S. [1]. A previous study investigating AF-related mortality in the U.S. during the first year of the pandemic found an increased mortality rate associated with AF, particularly among White men aged over 65 years [5]. It was previously reported that pre-existing atrial fibrillation (AF) increases the risk of in-hospital mortality in patients admitted for COVID-19 [6]. Furthermore, new-onset AF is an independent predictor of COVID-19 mortality [7]. However, there are limited data about the impact of the pandemic on AF-related mortality [5].

We aimed to investigate AF-related mortality trends before, during, and after the COVID-19 pandemic peak using a U.S. death certificate database stratified by sociodemographic factors such as age, sex, and geographical location.

## 2. Materials and Methods

We conducted a retrospective observational cohort study using data from the Centers for Disease Control and Prevention Wide-Ranging Online Data for Epidemiologic Research (CDC WONDER) Provisional Multiple Causes of Death (MCD) database [8]. This database includes county-level national mortality and population data spanning the years 2018–2024. The data were based on death certificates for U.S. residents between 1 January 2018 and 31 December 2023, which contain the main cause of death, a list of 20 contributing diagnoses, and demographic data. The number of deaths, crude death rates, age-adjusted mortality rates per 100,000 population, and 95% confidence intervals (95% CIs) for death rates can be obtained according to various factors. These factors include the cause of death (the four-digit codes of the International Statistical Classification of Diseases and Related Health Problems, Tenth Revision [ICD-10]), place of residence (nation, region, division, state, and county), age (single-year age groups, 5-year age groups, 10-year age groups and infant age groups), race (American Indian or Alaskan Native, Asian or Pacific Islander, Black or African American, White), sex and year. Age-adjusted mortality rates are calculated using the direct method based on data from the 2000 U.S. census as the standard population. The underlying cause of death is the disease or injury that initiated the series of events leading directly to death. The contributing cause of death is defined as any disease or injury that can be considered a contributing factor leading to death. AF diagnosis, as a main or contributing cause of death, was determined using ICD-10 codes I48xx. Patients with a COVID-19 diagnosis as an underling cause of death or contributing cause of death were identified using ICD-10 codes U07.1 and U09. Trends in AF-related mortality in the general population were analyzed using Joint Point software Version 5.2.0 (released 4 June 2024) to calculate the joint point regression. We stratified them by age, sex, race, and origin, and they were analyzed across the three chronological periods of the pandemic: pre-pandemic (2018–2019), pandemic peak (2020–2021), and pandemic decline (2022–2023) [9]. In addition, we calculated the burden of COVID-19-related mortality among this population. This study was waived from Institutional Review Board approval because the database from the CDC WONDER contains anonymized, publicly available data.

## 3. Results

We examined a total of 1,267,758 death certificates with AF as a contributing cause of death during the years 2018–2023. Among them, 79,669 (6.3%) death certificates had COVID-19 diagnosis as either the underlying or contributing cause of death. AF as the main cause of death was observed on 164,607 (13%) death certificates. Female patients accounted for 637,227 death cases (50.3%). Most of the population were older than 65 years (93.3%) and were of White ethnicity (90.3%) and non-Hispanic origin (Table 1).

**Figure 1:**
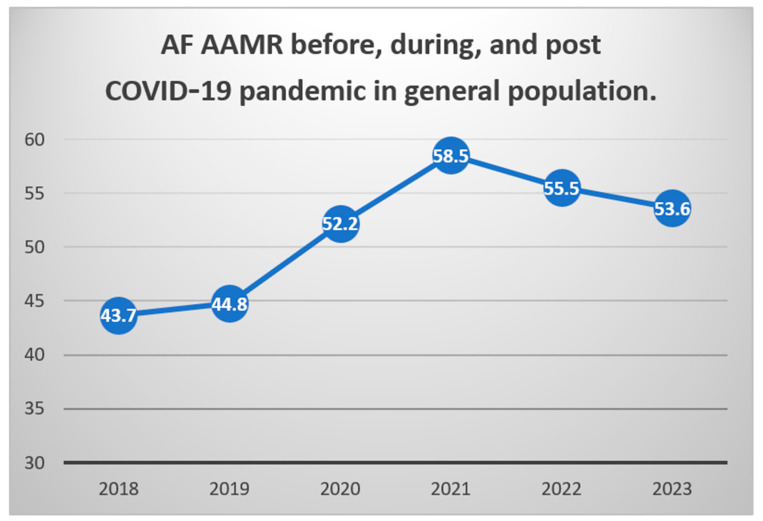
Annual AF-related mortality in the U.S. during 2018-2023. AF: pre-existing atrial fibrillation; AAMR: age-adjusted mortality rate per 100,000 population. For 95% confidence intervals, please refer to Supplementary Table S1.

In the general population, during the pandemic peak, there was a significant increase of 24.8% in the AF-related age-adjusted mortality rate (AF-AAMR) compared with the observed mortality rate during the pre-pandemic period (Table 2). We observed a significant trend of increased mortality between 2018 and 2021 (annual percentage change [APC]- 11%, 95% CIs: 8.3–14.9, *p* < 0.05) (Supplementary Figure S1). During the pandemic decline period, there was a non-significant decrease of 1.4% in the AF-related age-adjusted mortality rate (Figure 1). We observed a non-significant trend of decreasing mortality between 2021 and 2023 (APC −3.9%, 95% CIs: −7.6% to 0.3%, *p* > 0.05) (Supplementary Figure S1). 

### Trends by Sex, Age, and Race

In total, AF-related age-adjusted mortality was significantly higher among male vs. female patients (46.3 vs. 67 AF-AAMR, 95% CIs: 46.1–46.5 and 66.7–67.3, respectively). During the pandemic peak period, the increase in AF-related age-adjusted mortality was more pronounced in male patients (Table 2). However, during the pandemic decline period, the male group had an insignificant AF-related mortality decrease of 0.4%, while a significant decrease of 1.7% was observed in the female group (Table 2). Overall, the burden of AF-related mortality was higher among patients older than 65 years compared to younger individuals (355.1 vs. 6.5 per AF-AAMR, respectively). However, individuals younger than 65 years experienced a higher increase of 43.6% in AF-related deaths during the pandemic peak period compared to a 23.7% increase among patients older than 65 years (Table 2). Both age groups experienced a significant decrease in mortality during the pandemic decline period: 7.1% among the younger group versus 1.6% among the older group (Table 2).

All racial groups showed an increase in AF-related mortality during the pandemic peak period. African American patients experienced the most prominent relative mortality increase of 35.1% (Table 2). In contrast, while White AF patients experienced the highest burden of AF-related mortality (59.1 AF-AAMR per 100,000), they experienced the lowest prominent increase of 24.4% (Table 2). During the pandemic decline period, all racial groups, except for patients with multiracial origins, experienced a significant decrease in AF-related mortality. The most prominent decrease of 5.8% was observed among the American Indian group (Table 2). The increase in AF mortality among the Hispanic origin group was 39.5% during the COVID-19 peak years, and it was 24.51% in the non-Hispanic group (Table 2).

## 4. Discussion

Utilizing the CDC WONDER database, we analyzed a total of 1,267,758 AF-related deaths during the COVID-19 pandemic peak, the pandemic’s decline, and the two years before the pandemic. Of these, 6.3% were COVID-19-related deaths. During the pandemic peak period, a significant increase in AF-related mortality was observed, while during the pandemic decline period, a significant but less pronounced decrease in AF-related mortality was observed. These trends were consistent across subgroups, although males, African American individuals, and individuals of multiracial origins experienced a non-statistically significant decrease in AF-related mortality during the pandemic decline period. During the pandemic peak period, the most prominent increase in AF-related mortality was observed among male patients, among those younger than 65 years, and among individuals of African American and Hispanic descent.

The observed increase in AF-related mortality in our study is consistent with previous reports [10]. In a nationwide analysis of deaths due to cardiovascular causes, Wadhera et al. showed a significant increase in deaths caused by ischemic heart disease and hypertensive diseases during the initial phase of the COVID-19 pandemic [1]. Recently, an analysis of CDC WONDER revealed an increase in AF-related mortality during 2020 (pandemic onset) compared with previous years. However, comprehensive data regarding AF-related mortality during the pandemic peak (i.e., years 2020–2021) and decline (years 2022–2023) periods were not described [5].

There are several possible direct and indirect contributing factors that may explain our findings. Patients with AF tend to be older and have a higher burden of comorbidities, which subsequently puts them at risk of severe COVID-19 infection and mortality [11,12]. There is evidence that COVID-19 increases the risk of new-onset AF [13]. Rosenblat et al. reported that 5.4% of hospitalized patients with COVID-19 developed new-onset AF, which was associated with a 50% rate of in-hospital mortality [14].

Several pathophysiological mechanisms have been suggested to explain the previously reported increase in AF incidence during the COVID-19 pandemic, for example, hypoxemia that develops secondary to lung infection or the activation of the sympathetic system during COVID-19 infection [12], mechanisms which likely contributed to the higher AF-related mortality observed in our study. Severe COVID-19 infection may induce a cytokine storm [15]. Higher levels of circulating inflammatory markers, such as C-reactive protein, tumor necrosis factor, and interleukin (IL)-2, IL-6, and IL-8, were associated with new-onset AF [10]. Another suggested pathophysiological explanation is direct viral infection of the myocardium. The angiotensin-converting enzyme 2 receptor is expressed on myocardial microvascular pericytes and is assumed to play a role in the coronavirus’ entry into cells. The systemic inflammation induced by COVID-19, along with a significant influx of CD4+ T cells into cardiac tissues, may favor the development of AF [16]. 

An indirect effect of the COVID-19 pandemic may also explain the observed increase in AF-related mortality in the current analysis, in which only 6% of deaths were associated with COVID-19 infection itself. It is well established that other conditions, such as community-acquired pneumonia and sepsis, increase the risk of developing AF and of mortality, especially in patients with pre-existing AF [17]. These conditions can occur as secondary complications to COVID-19 and may contribute to the observed increase in AF-related mortality. Another indirect effect is the social distancing strategy. During the first months of the COVID-19 outbreak, individuals who were perceived as high-risk for severe COVID-19 were instructed by the Centers for Disease Control and Prevention (CDC) to avoid public gatherings [18]. This social distancing strategy led to a decrease in ambulatory clinic visits [19] and in elective procedures, specifically AF-related procedures such as AF ablations and cardioversions. The impact was more profound during the first wave of the pandemic, with a gradual return to pre-pandemic activity levels in subsequent waves [3,20].

### Trends by Demographic Groups

Patients younger than 65 years experienced a significant increase in AF-related mortality during the pandemic peak, nearly double that of the elderly group. Younger adults were prone to developing cardiac complications after receiving the COVID-19 mRNA vaccination, specifically pericarditis and myocarditis, associated with a higher risk of cardiac arrhythmia and death [21]. In addition, false-negative SARS-CoV-2 quantitative polymerase chain reaction (qPCR) test results were more likely among younger patients [22], resulting in a possible underestimation of the true burden of COVID-19 and its cardiac complications, including AF, among this population.

In our current analysis, male patients exhibited a more pronounced increase in AF-related mortality during the pandemic peak years compared to their female counterparts. Previous studies have demonstrated that males are more susceptible to developing severe COVID-19 and have a higher incidence of AF when they have the disease [23,24], which could potentially explain our observation.

Individuals of Hispanic origins had a higher relative increase in AF-related mortality (39%) compared to non-Hispanic individuals during the pandemic peak period. Black and African American individuals experienced the highest increase in AF-related mortality (35%). The observed findings appear to contradict the established notion that AF is more prevalent among White individuals [24]. This discrepancy may indicate the suboptimal management of AF within this population during the peak of the pandemic. Our findings emphasize the significant impact of COVID-19 on socially deprived minority groups, especially the Hispanic and African American population. Our findings correlate with previous studies which reported higher morbidity and mortality rates due to symptomatic COVID-19 among ethnic and racial minority groups [25,26]. Individuals belonging to minority groups are more likely to be at a low socioeconomic level [27]. As a result, they tend to reside in crowded neighborhoods, experience impaired healthcare services, and exhibit a higher rate of comorbidities such as diabetes mellitus, hypertension, and obesity [28,29]. In addition, they tend to experience structural racism, which could potentially interrupt their treatment [30]. These factors are associated with severe COVID-19 and worse AF-related outcomes [22,23].

During the pandemic’s decline years, we observed a total decrease in AF-related mortality of 1.45% across the entire cohort. A similar trend was observed in all subgroups. Our observation is in agreement with the reported decline in total mortality due to COVID-19 during the year 2022 [31], which may have been the result of global vaccination and natural exposure leading to less severe disease and improved outcomes [31]. However, novel COVID-19 vaccines were associated with peri-myocarditis development [32], potentially increasing the risk of new-onset AF [33]. Another possible explanation for the observed decrease in AF-related mortality is improved patient care, as the healthcare system adapted to the pandemic by utilizing remote medicine resources, including telemedicine [34]. One such project, called TeleCheck-AF, involved physicians delivering care to their patients through teleconsultations. This was supported by an on-demand photoplethysmography-based heart rate and rhythm monitoring app (FibriCheck^®^) [35]. However, outcome data regarding these approaches are currently lacking.

Our study has several limitations. Firstly, because the information was obtained from an online database of death certificate data, there is an element of misclassification bias, particularly given the potential for inaccuracies in coding the cause of death in the death certificate data. Secondly, the CDC WONDER database does not provide information on important contributory factors to cardiovascular mortality, such as differences in cardiovascular risk factor burden amongst individuals, prevalent cardiovascular diseases and their treatments, socioeconomic status, and healthcare access. Thirdly, there are no data about the duration of AF diagnosis, its types, or whether there is a history of treatments such as antiarrhythmic drugs or ablation therapy, which may all impact mortality outcomes. Fourthly, due to the CDC WONDER database’s constraints, we were unable to perform subgroup analyses according to AF as the primary or secondary cause of death. Fifthly, as reported in the meta-analysis, there was significant heterogeneity in the prevalence of AF among COVID-19 patients. This heterogeneity was influenced by factors such as geographical location, age, hypertension, and diabetes [36]. Unfortunately, these specific factors could not be evaluated through the database we used. Lastly, provisional data were used to analyze outcomes during the pandemic decline period; these data should be substantiated in future studies.

## 5. Conclusions

During the COVID-19 pandemic peak, a significant increase was observed in AF-related mortality compared with the pre-pandemic period. This increase was particularly pronounced among male patients, those younger than 65 years, and individuals of African American and Hispanic origins. Provisional data indicated that during the decline phase of the pandemic, there was a modest trend of decreased AF-related mortality.

Our findings suggest that for COVID-19 patients, as well as for patients in future healthcare crises, targeted healthcare policies such as widespread telemedicine and interventions like home hospitalization systems to identify AF may be necessary, given its potential impact on patients’ outcomes, while addressing disparities among different patient populations.

## Figures and Tables

**Table 1:** Unadjusted mortality data. COVID-19—coronavirus infection disease.

Unadjusted Mortality Data	Death Cases	Percentage of Population
General population	1,267,758	100.0%
COVID-19	79,669	6.3%
AF as main cause of death	164,607	13%
Female	637,227	50.3%
Aged ≥ 65	1,182,220	93.3%
White	1,144,262	90.3%
African American	82,819	6.5%
Asian	26,387	2.1%
American Indian	5445	0.4%
More than one race	4820	0.4%
Non-Hispanic origin	1,201,196	94.7%

**Table 2:** Age-adjusted AF-related mortality rates in the U.S., 2018–2023. AF: pre-existing atrial fibrillation; AAMR: age-adjusted mortality rate per 100,000 population. Pre-COVID-19: 2018–2019. During COVID-19 peak: 2020–2021. Post COVID-19 peak: 2022–2023. P.O.C: percentage of change.

	Pre-COVID-19	COVID-19 Peak	P.O.C	Post-COVID-19	P.O.C
General population	44.3 (44.1–44.4)	55.3 (55.1–55.4)	24.83%	54.5 (54.4–54.7)	−1.45%
Gender					
Male	52.2 (52.0–52.5)	67 (66.7–67.3)	28.4%	66.7 (66.5–67.0)	−0.45%
Female	38.2 (38.0–38.4)	46.3 (46.1–46.5)	21.20%	45.5 (45.3–45.7)	−1.73%
Age					
Aged < 65	3.9 (3.8–3.9)	5.6 (5.5–5.6)	43.59%	5.2 (5.2–5.4)	−7.14%
Aged ≥ 65	330.2 (329.1–331.3)	408.4 (407.2–409.6)	23.68%	404.1 (402.9–405.3)	−1.59%
Race					
American Indian	24.9 (23.5–26.2)	32.7 (31.2–34.2)	31.33%	30.8 (29.5–32.2)	−5.81%
Asian	19.1 (18.6–19.5)	24.2 (23.7–24.7)	26.70%	23.3 (22.8–23.7)	−3.72%
African American	28.8 (28.4–29.2)	38.9 (38.5–39.4)	35.07%	38.2 (38.3–39.1)	−1.80%
More than one race	17 (16.0–17.9)	21.9 (20.9–23.0)	28.82%	21.7 (20.7–22.7)	−0.91%
Native Hawaiian	37 (33.1–40.9)	47 (42.9–51.1)	27.03%	45.8 (42.1–49.8)	−2.34%
White	47.5 (47.3–47.6)	59.1 (59.0–59.3)	24.42%	58.50 (58.3–58.7)	−1.02%
Hispanic	23.3 (22.9–23.7)	32.5 (32.0–32.9)	39.48%	30 (29.6–30.4)	−7.69%
Non-Hispanic	46.1 (46.0–46.3)	57.4 (57.2–57.6)	24.51%	56.9 (56.7–57.0)	−0.9%

## Data Availability

The original contributions presented in the study are included in the article and Supplementary Material, further inquiries can be directed to the corresponding authors.

## Supplementary Materials

The following supporting information can be downloaded at: https://www.mdpi.com/article/10.3390/jcm13164813/s1.
