# Supplementary material for: The Trends in Atrial Fibrillation-Related Mortality before, during, and after the COVID-19 Pandemic Peak in the United States
Source: J Clin Med. 2024 Aug 15;13(16):4813. doi: 10.3390/jcm13164813 (PMC11355373; doi:10.3390/jcm13164813)
Supplement: Supplementary file 1 [file jcm-13-04813-s001.zip › jcm-3136038-supplementary.pdf]

Journal of
Clinical Medicine

MDPI

Supplementary Data

Supplementary Figure S1: Trends in AF-realated mortality 2018-2023 in the U.S

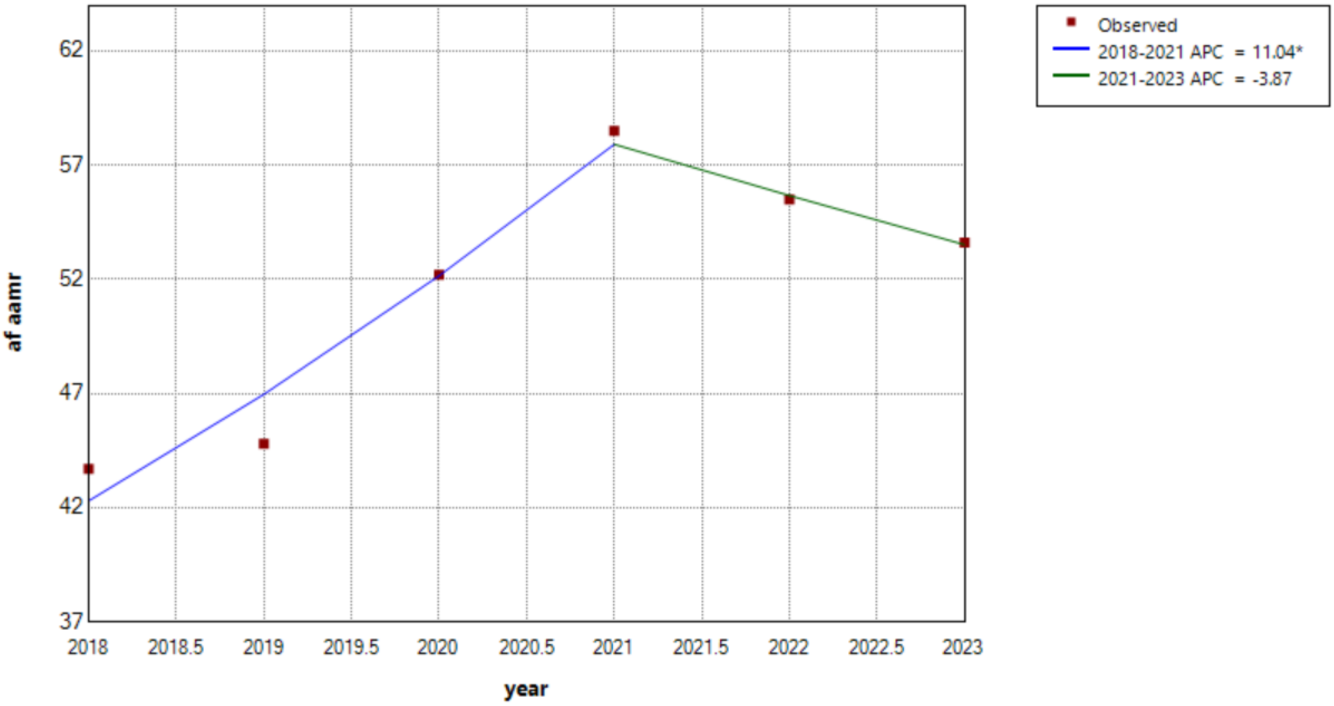

* Indicates that the Annual Percent Change (APC) is significantly different from zero at the alpha = 0.05 level.
-- Test Statistic and P-Value not available for the Empirical Quantile method.
Final Selected Model: 1 Joinpoint.

Year	AF-AAMR	95% C.I
**2018**	43.7	(43.5 - 43.9)
**2019**	44.8	(44.6 - 45.0)
**2020**	52.2	(52.0 - 52.4)
**2021**	58.5	(58.3 - 58.8)
**2022**	55.5	(55.3 - 55.7)
**2023**	53.6	(53.4 - 53.9)

Supplementary Table S1: Atrial Fibrillation related age-adjusted-mortality-rate (AMMR) and 95% coincidence intervals during the years 2018-2023. AF- pre-existing atrial fibrillation, AAMR- age-adjusted mortality rate per 100,000 population. C.I – confidence interval.
